# Improving the quality control of drinking water in Nicaragua through proficiency testing in a metrological multilateral cooperation project

**DOI:** 10.1038/s41598-021-96230-w

**Published:** 2021-08-19

**Authors:** Gabriel Molina-Castro, Jimmy Venegas-Padilla, Junette Molina-Marcia, Luciana Scarioni, Bryan Calderón-Jiménez

**Affiliations:** 1 Chemical Metrology Division, Costa Rican Metrology Laboratory, San Jose, Costa Rica; 2 By Order of Physikalisch-Technische Bundesanstalt, Braunschweig, Germany; 3 Laboratory of Natural Water, Research Center for Aquatic Resources of Nicaragua, Managua, Nicaragua

**Keywords:** Health care, Chemistry

## Abstract

The United Nations General Assembly explicitly recognized the human right to water and sanitation and acknowledged that drinking water is essential to the realization of all human rights in a 2010 resolution. Supporting and strengthening the quality infrastructure in countries throughout the world guarantees more reliable water quality analyses, thus reducing the risks to consumers’ health. The present paper describes a multilateral cooperation project developed in Nicaragua to improve the country's quality infrastructure and, in turn, the quality control of drinking water. The project was developed with the support of National Metrology Institutes (NMIs) from the Inter-American Metrology System (SIM), the Physikalisch Technische Bundesanstalt (PTB) and the participation of research institutes and laboratories in Nicaragua. Several mechanisms such as awareness seminars, workshops, metrological screenings, peer review of the laboratories’ quality systems, and organizing proficiency testing (PT) were used to successfully achieve the cooperation goal. As a result, technical infrastructure for the organization of PT rounds in Nicaragua was implemented to evaluate the relevant physicochemical parameters such as pH, chloride (Cl^−^), and nitrate (NO_3_^−^) in drinking water. The results from the PT rounds which took place during the two-year cooperation project showed substantial improvement in the performances of the participating laboratories, and therefore, in their measurement methods. Finally, this article shows how multilateral cooperation projects can strengthen the quality infrastructure, improving and ensuring the quality control of drinking water.

## Introduction

Water resources are a highly important component in every country due to their impact on health, the economy and the welfare of citizens. The United Nations General Assembly, through Resolution 64/292 on 28 July 2010^1^, explicitly recognized the human right to water and sanitation and acknowledged that drinking water is essential to the realization of all human rights. Many countries in Latin America are making considerable efforts to ensure the sustainability of water sources^2^. However, these countries must overcome some barriers that limit the correct use and maintenance of the water sources used for human consumption.

In the last decades, Nicaragua had established a legal framework to enhance the legislation and promotion of an integral management of water resources through key measures such as the general law of environment and natural resources^3^, the national policy on water resources^4^, and the general and national law of water and its regulations^5^. However, governmental and non-governmental institutions need to be strengthened to ensure that the technical challenges of drinking water can be met. For this aspect, it is still necessary to augment the technical capabilities of the quality infrastructure of Nicaragua, specifically to strengthen the measurements of the physicochemical parameters which are relied upon to ensure the quality of drinking water.

This discrepancy encouraged the National Metrology Institute of Germany (PTB, from its German acronym) and the Costa Rican Metrology Laboratory (LACOMET, from its Spanish acronym), with the collaboration of the Research Center for Aquatic Resources of Nicaragua (CIRA/UNAN, from its Spanish acronym) and the Nicaraguan National Metrology Laboratory (LANAMET, from its Spanish acronym), to develop a multilateral cooperation project to strengthen the metrological infrastructure of Nicaragua to assure the quality of drinking water. It was divided into two stages which aimed to improve the reliability of measurements and the evaluation of drinking water quality^6^. Both stages included the organization of workshops and training which presented an overview of the topics associated with two PTs that were developed in 2015 (PT-1) and 2016 (PT-2) for the measurement of pH, Cl^−^ and NO_3_^−^ in drinking water. The selection of these measurands is due to their presence in the first two most basic levels of control established in Nicaraguan legislation^5,7^, representing normal control parameters and basic indicators of unwanted substances in drinking water easily executable by any water quality control laboratory authorized.

Under the main question of whether international cooperation projects can improve the quality measurements of drinking water in developing countries through the use of proficiency testing, the present article describes the strategy and development of a metrological multilateral cooperation project designed for this purpose. It also illustrates the impact of workshops and training in the field of metrology, describes the results obtained in the PTs, and establishes the importance of infrastructure for PTs to measure improvement and to ensure the quality of the measurements involved.

## Materials and methods

**Figure 1:**
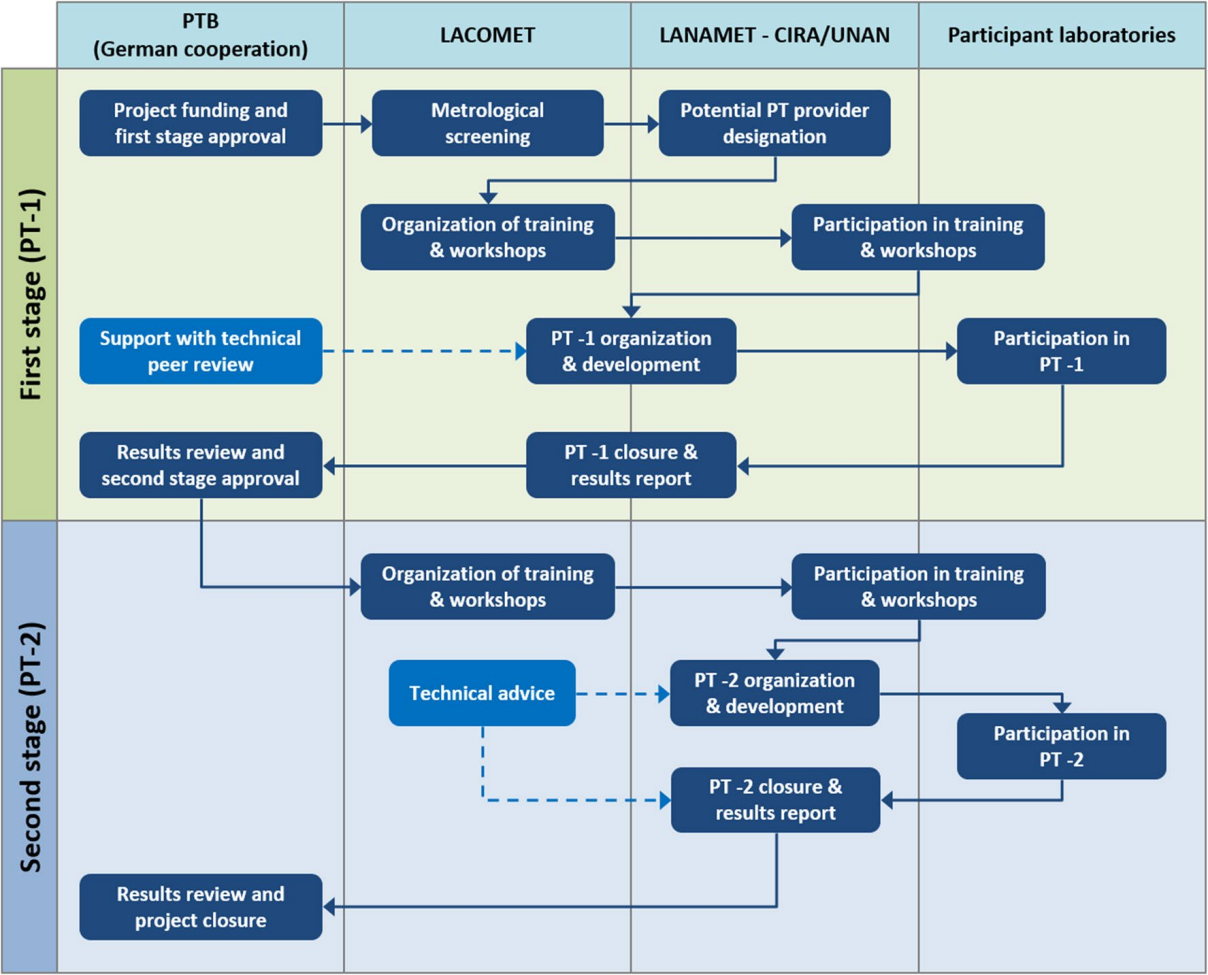
Development of the two-stage multilateral cooperation project.

A scheme of the cooperation project development is shown in Fig. 1. The main strategic aspects are briefly described below, where the Chemical Metrology Division of LACOMET acted as the technical leading institution and adviser for metrological screening, training, workshops, and PT organization. The cooperation project also involved the participation of ten testing laboratories from Nicaragua, all of which had the analytical capabilities to measure drinking water. The participation of these laboratories in the project stages is shown in Supplementary Table S1.

### Training, workshops, and other related activities

#### Metrological screening

Of the ten analytical laboratories enrolled as participants, only three were identified as potential PT providers based on their technical and analytical infrastructure. These laboratories were subjected to an exhaustive technical revision of ISO/IEC 17025^8^ requirements. After the screening process, only CIRA/UNAN-Managua and LANAMET were chosen as the potential PT providers.

#### Metrology workshops

Four workshops were developed to strengthen the technical aspects involved in the chemical measurements for drinking water and its uncertainty estimation^9^. The methods covered in these workshops, and in the project in general, included pH measurement using a glass electrode membrane^10^, Cl^−^ measurement using the potentiometry method^11^, and measurement of anions using ion chromatography (specifically Cl^−^ and NO_3_^−^)^12^. Despite the fact that there are currently new approaches to green chemistry that make it possible to trace physicochemical parameters of water^13^, the project sought to take advantage of the existing technical infrastructure in the participating laboratories. These analytical techniques were selected according to their capacities to avoid exclusions due to technological gaps, reducing implementations costs to ensure long-term sustainability to the project. The use of all these techniques is accepted according to the national legislation of Nicaragua^5,7^. All workshops were held at CIRA/UNAN-Managua facilities and covered both theoretical and practical aspects.

#### PT organization training

A training session at which the managerial and technical requirements for organizing PTs were to be described was developed based on ISO/IEC 17043^14^, ISO 13528^15^ and the IUPAC Harmonized Protocol^16^. Technical documents which were drafted and used by LACOMET served as examples. As a result, the selected potential PT providers prepared their own procedures, protocols, and invitation letters (among other materials) needed to organize a PT round.

#### External accompaniment (peer review)

To ensure the quality of the reference materials (RMs) used as PT items, all the procedures for the preparation, homogeneity, and stability evaluation as well as RM characterization used by LACOMET were subjected to international peer review by an expert from INMETRO (Brazil), based on ISO/IEC 17043 and ISO Guide 34 requirements^17^. During the reviewer’s visit to LACOMET, minor improvements were suggested, including more detailed planning of the reference material production, extensive validation of spreadsheets, and a road map to control the production and filling processes.

#### RM preparation training

Specific training involving all the technical aspects related to gravimetric preparation of the RMs, homogeneity assessment and experimental homogeneity study, value assignment, and stability studies^15,18^ was developed at LACOMET’s facilities in Costa Rica. A technical delegation from Nicaragua made up of CIRA/UNAN-Managua and LANAMET staff attended this training.

### Chemicals, reagents, and RMs

All solutions used were prepared using high-purity deionized type I water (resistivity ≥ 18 MΩ·cm at 25 °C). Reagent grade ACS sodium chloride (NaCl; Sigma Aldrich, USA), sodium nitrate (NaNO_3_; Sigma Aldrich, USA), and sodium tetraborate decahydrate (Na_2_B_4_O_7_·10H_2_O; Merck, USA) were used to prepare RM batches of Cl^−^, NO_3_^−^ and alkaline pH for the PT-1. In the case of the PT-2, NaCl (Sigma Aldrich, high purity grade), potassium nitrate (KNO_3_, Sigma Aldrich, high purity grade), and potassium hydrogen phthalate (C_8_H_5_KO_4_; Merck, USA) were used to prepare the RM batches for Cl^−^, NO_3_^−^ and acid pH, respectively.

Cl^−^ and NO_3_^−^ standard solutions and calibration solutions were prepared daily and gravimetrically from a (998.7 ± 3.0) mg kg^−1^ standard reference material of Cl^−^ (SRM 3182, NIST) and a (999.8 ± 2.3) mg kg^−1^ standard reference material of NO_3_^−^ (SRM 3185, NIST). Certified buffer solutions with pH values of 4.01, 7.00, and 10.01 with an associated expanded uncertainty of 0.02 at 25 °C were used to calibrate and assess the performance of the pH meter (Mettler Toledo, Switzerland).

### Materials and equipment for preparation of RMs

High-density polyethylene (HDPE) containers with a maximum volume of 8 L and glass containers with a maximum volume of 11 L were used to prepare RM batches for PT-1 and PT-2, respectively. 125 mL HDPE and 250 mL polypropylene bottles were used as containers for all the prepared RMs. Aluminium foil zip lock bags were used for packaging and to avoid solvent transpiration from the prepared RMs. Model XPE6003SD5 (Mettler Toledo, Switzerland), CCE60K3 (Sartorius, Germany) and CCE1005 (Sartorius, Germany) balances were used for gravimetric preparation of batches and gravimetric dilutions of the standards solutions. Finally, a Milli-Q A10 water purification system (Millipore, Paris, France) was used to supply the high purity type I water. A summary of the gravimetric preparation of the RM batches is shown in Supplementary Table S2.

### Methods and equipment for the characterization of the prepared RMs

The ISO 10304–1 method^12^ was used to quantify the concentration of NO_3_^−^ and Cl^−^, including homogeneity and stability measurements. Specifically, an ion chromatograph (IC) (850 Professional, Metrohm, Switzerland) with a conductivity detector and polyvinyl alcohol/quaternary ammonium column (Metrosep A Supp 5-150/4.0, Metrohm, Switzerland) was used. Details about operating conditions used in the IC system are shown in Supplementary Table S3. Standard reference materials—SRM 3185 and SRM 3182—provided metrological traceability to these measurements.

The ISO 10523 method^10^ was used to perform the pH measurements, including homogeneity and stability measurements. Calibration of pH meter and uncertainty estimation were performed using a bracketing procedure as recommended by the IUPAC^19^. All measurements were carried out at (25 ± 1) °C. A pH meter (781 pH/Ion digital, Metrohm, Switzerland) with automatic temperature compensation (ATC), an automatic stirring system (801, Metrohm, Switzerland) and a glass electrode with a Pt-1000 temperature sensor (Unitrode, Metrohm, Switzerland) were used to measure pH. The certified buffer solutions mentioned above provided metrological traceability.

### Statistical design and data evaluation

Both PTs were based on a simultaneous scheme^14^, expecting for unimodal, relatively symmetric distribution of quantitative results^16^. Since the expected number of participants was reduced, the use of consensus values was avoided for the performance evaluation^20^. Participating laboratories reported measurement uncertainty for description purposes^9^, although it was not included in the evaluation. For all the calculations, data processing, and statistical evaluation, Microsoft Excel 2010 (14.0.7177.5000)^21^ and R (version 3.1.1), a free environment for statistical computing^22^, were used.

#### Value assignment and traceability sources

For the value assignment in the RMs (*x*_*pt*_), up to two sources of information were considered: the gravimetric preparation mean (*x*_*grav*_) and the homogeneity study mean (*x*_*hom*_). The choice of this strategy should be understood as a practical approach for the purpose of proficiency testing and not for certification of the RMs. Although gravimetric values represent a better estimate of the assigned value according to ISO Guide 35^18,23^, their traceability chains were broken because of the absence of certified purities. To avoid possible biases, gravimetric values were complemented with homogeneity results, which do have complete metrological traceability through the certified reference materials described above. It should be highlighted that combining results from analytical methods with a gravimetric method to produce a single value is a strategy widely used both for proficiency testing and RM certification purposes^23,24^. Also, this approach produces higher uncertainties, which were evaluated as discussed below. To ensure validity of this strategy, agreement of *x*_*grav*_ and *x*_*hom*_ was evaluated by comparing uncertainty intervals and estimating the effective unilateral degrees of equivalence with a DerSimonian-Laird approach, using the NIST Consensus Builder software^25^ in a post hoc analysis for this paper.

For Cl^−^ and NO_3_^−^ concentration values, Eq. (1) was used to combine *x*_*grav*_ and *x*_*hom*_ in agreement and its combined measurement uncertainty was estimated using Eq. (2), a simple lineal combination of both uncertainty components^26^ chosen as a practical approach for the purpose of proficiency testing. For pH values, *x*_*hom*_ was used as the RM assigned value since *x*_*grav*_ does not correspond to an accurate estimation of pH. Likewise, the combined measurement uncertainty was estimated using a lineal combination of known or suspected uncertainty components.

1 $$x_{pt} = \frac{1}{n}\mathop \sum \limits_{i = 1}^{n} x_{i} = \frac{{x_{grav} + x_{hom} }}{2}$$

2 $$u\left( {x_{pt} } \right) = \sqrt {u_{grav}^{2} + u_{hom}^{2} }$$

To discard the influence of uncertainty overestimation and reduce the risk of incorrect performance evaluation due to inaccuracy of the assigned values, estimated standard uncertainties were evaluated against the standard deviation for proficiency assessment (*σ*_*pt*_) using Eq. (3).^15^

3 $$u\left( {x_{pt} } \right) \le 0,3\sigma_{pt}$$

Metrological traceability to the SI for the assigned values was partially achieved by the calibration with certified reference materials mentioned above, together with a comprehensive uncertainty budget estimation. However, this was not considered a limitation for the project due to the purpose of the RM for proficiency testing, whose reference standard ^14^ does not require the need for traceability in the assigned values for the performance evaluation of participants.

#### Standard deviations for proficiency assessment

For Cl^−^ and NO_3_^−^, *σ*_*pt*_ was estimated using a modified Horwitz general model for the reproducibility of analytical methods^15,16,27^ as a conservative strategy due to the limited number of laboratories (*n* < 20) and the lack of certainty a priori about consensus in its results. In the case of pH, due to the physicochemical nature of this measurand and the same reasons described before, *σ*_*pt*_ was estimated using historical values from previous PT rounds organized by LACOMET^28,29^ in which the number and type of participants were similar.

#### Data processing and performance evaluation

Participants’ results and their uncertainties were compared with the assigned value and its uncertainty to identify possible uncertainty overestimation or underestimation and to establish improvement strategies. Before performance evaluation, a visual blunder removal was made by expert criteria. The removal was followed by a statistical distribution revision of the remaining results and a calculation of their robust estimators (robust mean x^*^ and its uncertainty *u*(x^*^)) using Algorithm A^15^. Those robust results were then compared with the assigned values of RMs using Eq. (4), looking for consistency and to verify the statistical design assumptions^15^.

4 $$\left| {x_{pt} - x^{*} } \right| \le 2\sqrt {u^{2} \left( {x_{pt} } \right) + u^{2} \left( {x^{*} } \right) }$$

All the participants’ results were evaluated using a *z*-score as the performance statistic^14,15^, calculated in Eq. (5), where *x*_*i*_ is the reported result of the *i*^*th*^ participant.

5 $$z_{i} = \frac{{\left( {x_{i} - x_{pt} } \right)}}{{\sigma_{pt} }}$$

In cases where Eq. (3) did not meet the threshold, the standard uncertainty of the assigned value was incorporated in the evaluation using *z’*-score as the performance statistic, calculated in Eq. (6).

6 $$z^{\prime}_{i} = \frac{{x_{i} - x_{pt} }}{{\sqrt {\sigma_{pt}^{2} + u^{2} \left( {x_{pt} } \right)} }}$$

The conventional criteria for scores interpretation are reported in references^15,16^.

### Organization of PTs for drinking water in Nicaragua

**Figure 2:**
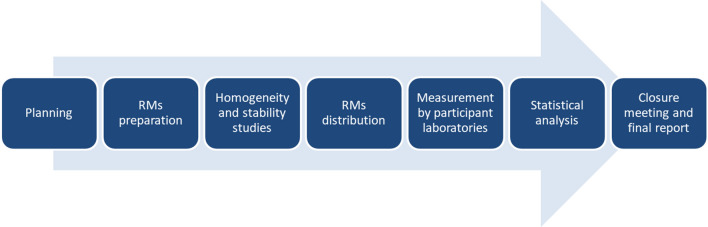
Process for the organization of PTs carried out in the multilateral cooperation project.

Because Nicaragua did not have a formal national infrastructure to develop and organize PTs in drinking water so far, the project established a "follow-up" strategy. In the first stage, the approach addressed the organization and development of PT-1 as a joint effort between LACOMET, CIRA/UNAN-Managua and LANAMET, so that the potential PT provider "learned by doing". In the second stage, independent organization and development of PT-2 was carried out by the potential PT provider with minor assistance which was limited to technical aspects. The general process used for the organization and development of both PTs in this cooperation project is shown in Fig. 2.

#### Planning of PT-1 and PT-2, and RMs preparation

The main goal of PT-1 was to establish the baseline of participating laboratories’ measurements and evaluate their performances for the determination of pH, Cl^−^ and NO_3_^−^ in drinking water. According to the “follow-up” strategy, PT-1 planning was led by LACOMET with an active participation from CIRA/UNAN-Managua and LANAMET staff. All of the necessary documents were drafted by CIRA/UNAN-Managua and LANAMET as result of the PT organization training, and RMs were prepared in LACOMET as mentioned above. On the other hand, the main goal of PT-2 was to evaluate the performance and improvement of the participating laboratories after the first stage, in order to measure the progress and success of the project. The same measurands were selected, but new nominal values were used, ensuring that they were adequate for the participating laboratories’ capabilities and the requested levels in the national regulations of Nicaragua^28^ (as shown in Supplementary Table S4). The PT-2 planning process was carried out by CIRA/UNAN-Managua and LANAMET; minor technical advice was provided by LACOMET. RMs were again prepared at LACOMET, and staff from CIRA/UNAN-Managua and LANAMET took part in that process during the training session previously mentioned.

#### Homogeneity and stability studies

In both PTs, the prepared RMs were studied at LACOMET. Homogeneity was assessed for the analysis of ten randomly selected samples from each prepared batch before the distribution of RMs among the participants. These samples were tested in duplicate using the procedures described above. A trend analysis was performed on the data^15,18^, and the presence of samples with outlier variances was evaluated using the Cochran test^30,31^. Finally, between-sample standard deviation (*s*_*s*_) was estimated and compared with the corresponding *σ*_*pt*_, as recommended in ISO 13528. Stability evaluation was performed by LACOMET through the analysis of two additional randomly selected samples from each prepared batch. To test the stability during shipping and storage, these samples were sent to Nicaragua with all the other RMs and then returned to Costa Rica. The samples were tested in triplicate, using the measurement instruments and methods described above. The homogeneity mean (*x*_*hom*_) was compared with the stability mean (*x*_*stb*_), and the difference was compared with the corresponding *σ*_*pt*_, as recommended on ISO 13528.

#### Distribution of RMs and measurement by participating laboratories

In both PTs, the prepared RMs were sent from Costa Rica to the potential PT provider in Nicaragua for their distribution among the participating laboratories. The participating laboratories reported their results to LACOMET in PT-1 and to CIRA/UNAN-Managua in PT-2 within one month for data processing and statistical evaluation.

#### Statistical analysis of both PTs

Statistical analysis was performed by LACOMET in PT-1 and by CIRA/UNAN-Managua and LANAMET in PT-2. Limited advising was provided by LACOMET during PT-2. This statistical analysis included the estimation of the RMs’ assigned values and *σ*_*pt*_, data processing, and performance evaluations of participants’ results, as mentioned above.

#### Closure meeting and final report

For both PTs, once the results were processed, CIRA/UNAN-Managua and LANAMET, with the support of LACOMET, prepared the PT final reports with the statistical evaluation results. Closure meetings with the participating laboratories were held to present the main results and conclusions.

### Ethical approval and informed consent

The authors declare no human or animal subjects were included and no informed consent was needed in this research.

## Results and discussion

### Technical discussion of the PTs

#### Evaluation of the prepared RMs, assigned values, and deviations for proficiency assessment

The fulfilment of the chosen criteria described above indicated that the design of the PT was appropriate for the purposes and assumptions. The main results of the studies and the evaluation of their respective criteria are shown in Table 1. For Cl^−^ and NO_3_^−^, agreement between *x*_*grav*_ and *x*_*hom*_ values was found in all cases. Details about these agreements are shown in Supplementary Fig. S1.

**Table 1:** Main results of the project design and evaluation criteria for PTs in the cooperation project.

Variables and criteria	pH (1)		Cl^−^ (mg L^−1^)		NO_3_^−^ (mg L^−1^)	
	PT-1	PT-2	PT-1	PT-2	PT-1	PT-2
*x*_*pt*_	9.17	4.03	74.0	86.0	46.8	30.4
*σ*_*pt*_	0.06	0.06	6.2	7.0	4.2	2.9
*u*(*x*_*pt*_)	0.015	0.025	0.2	0.6	0.25	0.15
Uncertainty^a^	✓	❌	✓	✓	✓	✓
*s*_*s*_	0.003	0.00	0.34	0.00	0.0	0.0
Homogeneity^b^	✓	✓	✓	✓	✓	✓
| *x*_*hom*_ – *x*_*stb*_ |	0.006	0.01	0.05	0.12	0.2	0.05
Stability^b^	✓	✓	✓	✓	✓	✓
x*	9.06	4.00	74.6	86.2	47.9	32.6
*u*(x*)	0.06	0.025	2.5	1.4	5.8	2.0
Consistency^c^	✓	✓	✓	✓	✓	✓

✓, Meets criterion; ❌, Does not meet criterion.

^a^According to Eq. (3).

^b^According to ISO 13528:2015.

^c^According to Eq. (4).

It can be noted that, for NO_3_^−^ and Cl^−^ in drinking water, the assigned values maintained similar levels in both PT-1 and PT-2 since these values were chosen to be within the ranges of the measurement capabilities of the participants. In the case of pH value, a different measurand level was chosen for each PT so that participants measured the pH on positive and negative potentials. When analyzing uncertainties related to the assigned value, Table 1 shows that all standard uncertainties of the assigned values *u*(*x*_*pt*_) were under 1%. In the case pH determination, the differences between uncertainties were attributed to larger variations in the measurement process of the certified reference materials (CRMs) – most likely due to measurement repeatability.

Since it was not expected that the dispersion of the participants’ results would increase or decrease with the pH level, a fixed *σ*_*pt*_ value of 0.06 was considered fit for purpose for both levels. In the case of NO_3_^−^ and Cl^−^, an opposite situation resulted in the application of the Horwitz model, since a proportional dispersion to the measurand level was expected. Specifically, *σ*_*pt*_ values for NO_3_^−^ concentration were only approximately 9% of their assigned value, while *σ*_*pt*_ values for Cl^−^ concentration were approximately 8%. It should be noted that the Horwitz model predicts the reproducibility variation expected for a certain concentration value^16^, which at first is expected to be relatively high, but since concentration nominal values were in the order of 0.01%, variations tend to be low due to the function’s “trumpet” shape^27^. In all cases but one, *u*(*x*_*pt*_) met the limiting criterion regarding *σ*_*pt*_, which implies that the uncertainties estimated with the practical approach of Eq. (2) were fit for the statistical design proposed in general. The actions that were taken to avoid incorrect performance evaluation due to the incompliance of this criterion are discussed below.

Table 1 also shows that, in all cases, *s*_*s*_ satisfied the criterion for sufficient homogeneity. Thus, it can be affirmed that the RMs resulted in sufficient homogeneity for the PTs purposes, and therefore, it was correct to assume that heterogeneity did not affect the evaluation of the participants. No trends by sample preparation or measurement order were detected, as shown in Supplementary Fig. S2. Moreover, the application of the Cochran test on homogeneity studies data only revealed the presence of outlier samples with substantially greater variances for NO_3_^−^ and Cl^−^ in PT-2. The cause analysis concluded that instrumental low repeatability was due to electrical fluctuations and the use of a contaminated sample container by mistake. These data were therefore discarded in the first stages of the respective studies. Finally, differences in time met the criterion for sufficient stability in all cases. Thus, it can be affirmed that the prepared RMs were sufficiently stable for the PT purposes, and therefore, it was correct to assume that instability did not affect the participants’ evaluation. The stability study results of both PTs are shown in Supplementary Fig. S3.

#### Data processing and analysis of participant’ results

**Figure 3:**
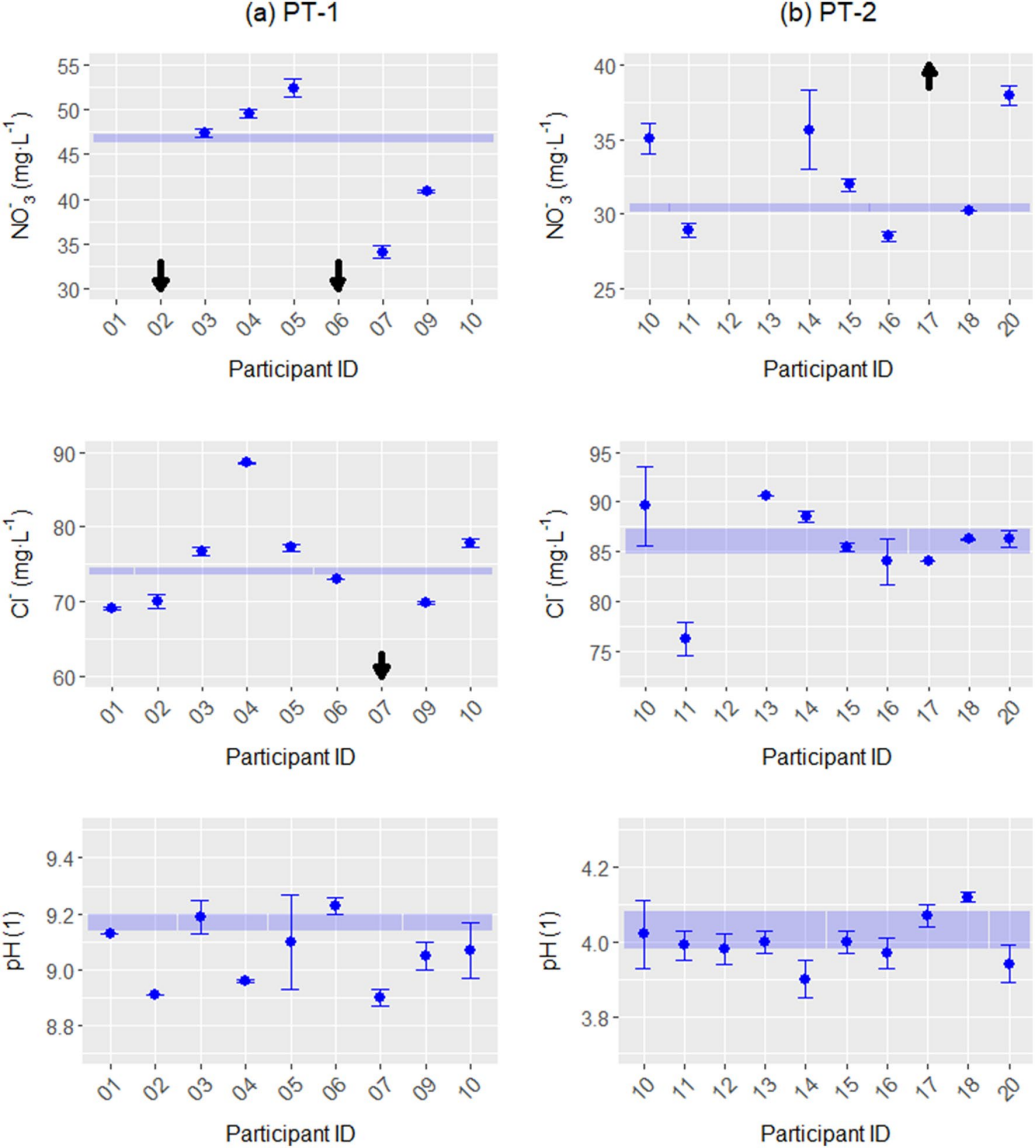
Comparison of the participants’ results and their reported expanded uncertainties with the assigned values and their estimated expanded uncertainties for columns (**a**) PT-1 RMs and (**b**) PT-2 RMs (arrows represent values outside axis limit).

The first stage of data processing and analysis consisted of comparing the results of the participants and their reported uncertainties with the assigned values and their estimated uncertainties for each measurand as shown in Fig. 3. In PT-1, participants reported a similar number of results above and below the assigned values for NO_3_^−^ and Cl^−^, and possible underestimations of uncertainties were observed in their results. In the case of pH, a larger number of results below the assigned value were observed, and the data presented both possible underestimations and overestimations of the uncertainties. It should be mentioned that the information derived from this analysis served as highly valuable input for the planning of the 2nd stage of the cooperation—particularly to narrow down the workshop topics. In PT-2, participants reported a similar number of results above and below the assigned values for pH and Cl^−^, while a larger number of results above the assigned value were observed for NO_3_^−^. Again, possible underestimations of uncertainties were observed in NO_3_^−^ and Cl^−^ measurements, accompanied by one or two overestimated cases. In the case of pH, the modifications of the procedure incorporated in PT-2 led to a substantial reduction of the variability of the measurements, reporting adequate uncertainty estimations for most cases with only one clear underestimation.

A visual blunder removal was performed on the participants’ results after the first stage of data processing^15^. In PT-1, three blunders were identified and removed from the data—two for NO_3_^−^ and one for Cl^−^. No blunders were identified for the pH. In PT-2, only one blunder was identified and removed from the data for NO_3_^−^ results; no blunders were identified for the Cl^−^ and pH results. Following removal of the blunders, the remaining data were plotted as a histogram and compared to the RM assigned values and the expanded uncertainty (*k* = 2) for each measurand (see Supplementary Fig. S4). Relatively symmetric unimodal distributions were obtained in all cases for PT-1 with a mode that was very close to the assigned values for NO_3_^−^ and Cl^−^. In PT-2, relatively symmetric unimodal distributions were obtained for Cl^−^ and pH with modes that were very close to the assigned values in both cases. In the case of NO_3_^−^, a relatively uniform distribution of the results was obtained with no clear mode, but with the assigned value within the observed distribution. With these results, the expected distributions were confirmed despite the relatively small number of participants. Additionally, consistency was found between the robust estimators of participants’ results and assigned values for both PT rounds. Thus, these results clearly demonstrate that the expected distribution is fit for the evaluation of the participants.

#### Performance evaluation and involvement of the participating laboratories

The interest in a standardized evaluation for all the measurands justified the selection of *z*- and *z’*-scores. The performance of the participants was assessed using *z*-scores in all the cases except in pH for PT-2 where *z*’-scores were used due to the possible influence of *u*(*x*_*pt*_). The estimated scores were classified using the conventional criteria for their interpretation^15,16^. Table 2 shows the performance evaluation results of PT-1 and PT-2. It should be noted that participant 8 decided not to report the results for PT-1 due to a management decision.

**Table 2:**
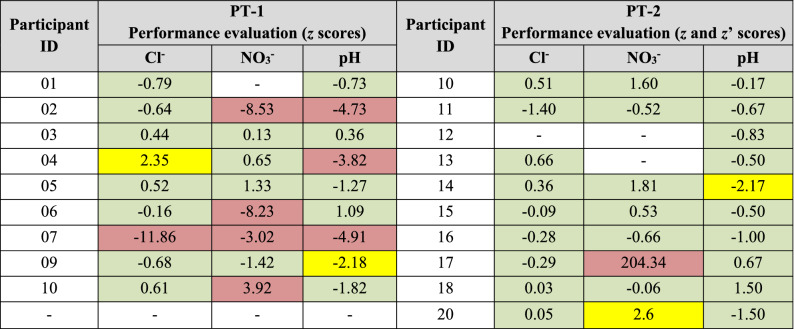
Performance evaluation results for PT-1 and PT-2 (green = acceptable results, yellow = questionable results and red = unacceptable results).

As shown in Table 2, Cl^−^ represented the best performances in PT-1: 78% of performance evaluations qualified as acceptable and only 11% of performance evaluations qualified as unacceptable. The performance for pH followed with 56% rate of acceptable performances and a 33% rate of unacceptable performances. Both the acceptable and unacceptable performances obtained for NO_3_^−^ were 50%. Better results were obtained in PT-2. Again, Cl^−^ represented the best performances with a 100% rate of acceptable results. The percentage for pH followed with 90% acceptable performances and no unacceptable performances. The majority of the NO_3_^−^ performances (75%) were acceptable and only 12.5% of the performances were unacceptable.

**Figure 4:**
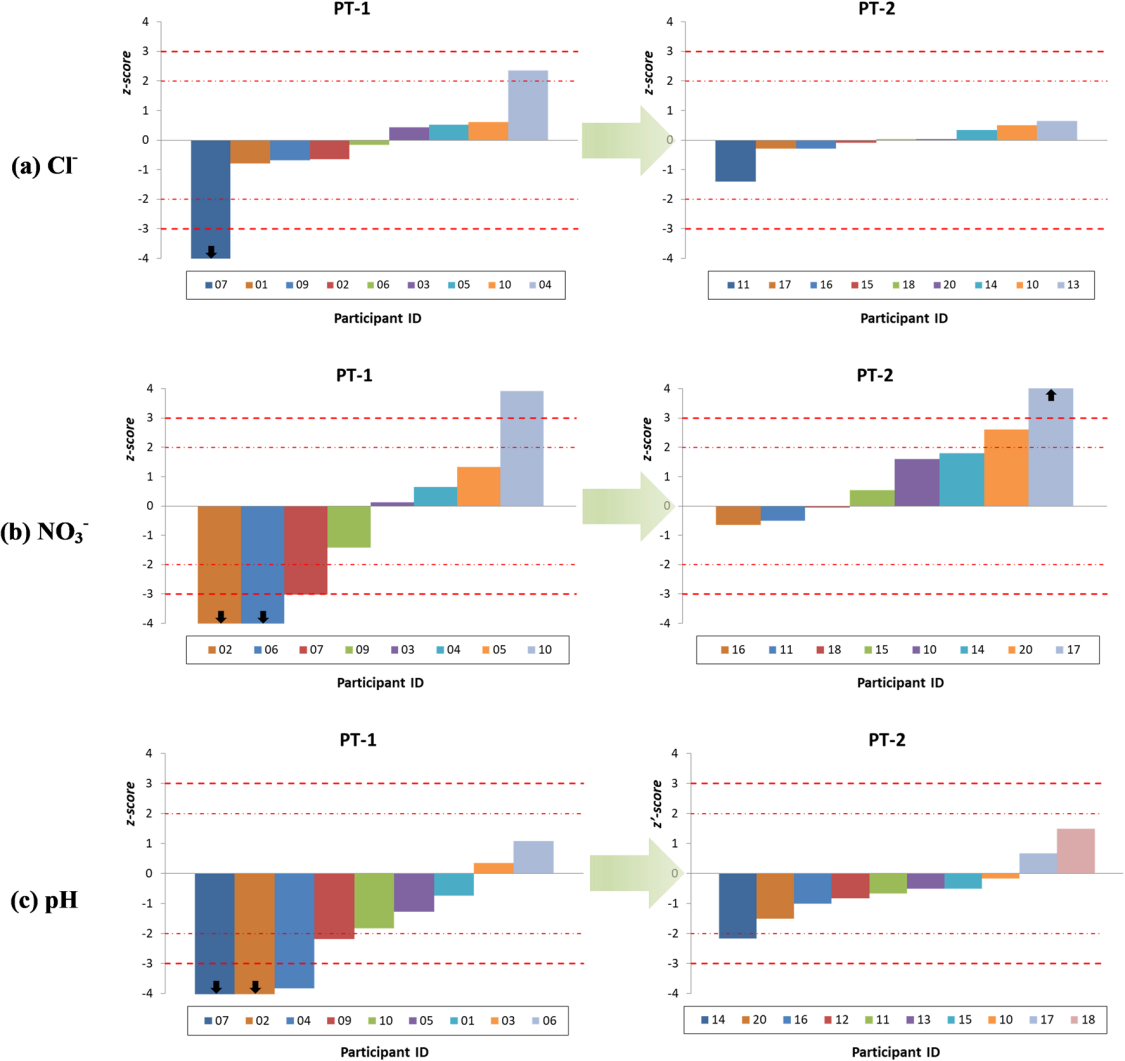
Improvement in performances of participants between PT-1 and PT-2 for rows (**a**) Cl^−^, (**b**) NO_3_^−^ and (**c**) pH (arrows represent values outside axis limit).

Accordingly, an important improvement in performance evaluations of the participants’ results was observed between PT-1 and PT-2. This situation was expected, since the results of PT-1 were used to develop a specific workshop related to measurement methods before PT-2. Figure 4 shows the improvement in performances evaluated between PT-1 and PT-2.

### Impact of the cooperation project on the quality infrastructure of Nicaragua

Besides the performance improvement of the participants, an implied improvement in the applied measurement methods can be concluded. This is one of the major impacts of the project, given that routine tests carried out by the participating laboratories are focused on assessing the potability of the water consumed in Nicaragua. In this way, by improving the performance of measurement methods, including uncertainty estimations, the project strengthens the metrological infrastructure that ensures the quality of drinking water, using proficiency testing as a key tool for measuring such improvement.

Additionally, the participating testing laboratories had the opportunity to access custom specialized training in physicochemical analysis in drinking water through the project, giving them the chance to meet the personnel training requirements of the ISO/IEC 17025 standard. Also, laboratories had the opportunity not only to evaluate and to validate their measurement methods and uncertainty estimations, but also to evaluate the effectiveness of training workshops and personnel improvement through a two-year external quality assurance programme. This, in turn, served as additional evidence for compliance with the technical requirements of the ISO/IEC 17025 standard. The fulfilment of the ISO/IEC 17025 requirements by testing laboratories also strengthens the Nicaraguan metrological infrastructure, providing laboratories with objective evidence of technical competence that can be submitted for an accreditation process, improving quality and confidence of measurement results in the country.

Last but not least, according to the National Accreditation Office of Nicaragua (ONA, by its Spanish acronym), two of the participating laboratories took advantage of their acceptable results in PT-1 and PT-2 for accreditation purposes and were able to demonstrate compliance with both the applicable requirements of the ISO/IEC 17025 standard and accreditation policies. This is another key impact of the project: as a national proficiency testing scheme was developed that met the requirements of the accrediting body, the national laboratories could demonstrate their technical competence without having to incur additional expenses associated with international proficiency testing which is sought in the absence of national PT schemes. In order to maintain this national benefit, a third independent PT round^32^ was organized by CIRA/UNAN-Managua and LANAMET one year after PT-2. Again, this PT was sponsored by a bilateral project of PTB with Nicaragua and it received some technical advice from LACOMET which was limited to statistical analysis assistance. The approach of this new independent PT included conductivity and hardness tests in drinking water, and featured the participation of many laboratories—including the same testing laboratories of the project as well as several Costa Rican testing laboratories that were invited by LACOMET. After the success of this new PT round, CIRA/UNAN-Managua staff, based on the know-how acquired from the cooperation, developed the research proposal “Evaluation of Environmental Measurements through a Proficiency Testing for Testing Laboratories in Nicaragua” which was approved for Funds for Research Projects of the National Autonomous University of Nicaragua (UNAN). The short-term goal of the new proposal is to evaluate the competence of laboratories participating in the measurement of physicochemical parameters of drinking water (using cation concentration and electrolytic conductivity as measurands). The long-term goal is to consolidate CIRA/UNAN-Managua, in conjunction with LANAMET, as the first accredited PT provider under the ISO/IEC 17043 standard in Nicaragua. The execution of this proposal will allow to Nicaragua to have a PT provider that will ensure the maintenance of the quality of drinking water measurements, and therefore, ensure the quality of the water consumed by its population.

## Conclusions and outlook

In this project, it was shown that multilateral cooperation projects in the field of metrology can be successful and that using proficiency testing to not only measure the improvement and the impact associated with the project, but also to ensure the maintenance of project benefits like the continuous control of measurement quality is important. In this way, the establishment of a PT program such as the one achieved with this project makes it possible to demonstrate the improvement and continuous control of the metrological infrastructure that guarantees the potability of the water consumed in a country.

The lessons learned from the project include the observation that rigorous attention must be paid to ensure the involvement of different actors of the national quality system (for example accreditation bodies, standardization institutes and national metrology institutes, among others) before starting a cooperation project. From a metrological point of view, it is also necessary to carry out a mapping of technical capacities in terms of infrastructure and human resources to ensure that enough capabilities are developed to provide good measurements and solid PT rounds. Future work in this field could be directed towards the use of new technologies that incorporate a greener chemistry alternative without losing the reliability of the results and their traceability to the SI.

As a result of this cooperation, all the expertise and skills that were acquired at the workshops and PTs enable CIRA/UNAN-Managua and LANAMET to promote the necessary technical bases and infrastructure conditions to establish, in the medium term, a dedicated working group that will organize and develop PTs in accordance with ISO/IEC 17043. As an initial PT provider in Nicaragua, CIRA/UNAN-Managua and LANAMET must become a leading player in the development of their national quality system through accreditation, facilitating the conditions for new PT providers to arise.

Finally, the global metrological community (especially regional projects and national metrology institutes) is invited to consider this particular project as successful and replicate its working methodology in order to improve national quality systems and the quality control of drinking water in other countries around the world.

## Supplementary Information

Supplementary Information.

## Data Availability

The datasets analyzed during the current study are available from the corresponding author on reasonable request.

## Abbreviations and acronyms

ACS: American Chemical Society
ATC: Automatic temperature compensation
CIRA/UNAN: Research Center for Aquatic Resources of Nicaragua
CRM: Certified reference material
HDPE: High-density polyethylene
IC: Ion chromatography
IEC: International Electrotechnical Commission
INMETRO: National Institute of Metrology, Quality and Technology of Brazil
ISO: International Organization for Standardization
IUPAC: International Union of Pure and Applied Chemistry
LACOMET: Costa Rican Metrology Laboratory
LANAMET: Nicaraguan National Metrology Laboratory
NIST: National Institute of Standards and Technology of the USA
NMI: National Metrology Institutes
ONA: National Accreditation Office of Nicaragua
PT: Proficiency testing
PTB: Physikalisch Technische Bundesanstalt
RM: Reference material
SI: International System of Units
SIM: Inter-American Metrology System
SRM: Standard reference material
UNAN: National Autonomous University of Nicaragua

## Chemical compounds, ions and other

C_8_H_5_KO_4_: Potassium hydrogen phthalate
Cl^−^: Chloride ion
Na_2_B_4_O_7_·10H_2_O: Sodium tetraborate decahydrate
NaCl: Sodium chloride
NaNO_3_: Sodium nitrate
NO_3_^−^: Nitrate ion
KNO_3_: Potassium nitrate
pH: Potential of hydrogen

## Variables

*n*: Number of replicates, laboratories or elements
*s*_*s*_: Between-sample standard deviation
*u*: Standard uncertainty
*x**: Robust mean
*x*_*grav*_: Gravimetric preparation mean
*x*_*hom*_: Homogeneity study mean
*x*_*i*_: Reported result of the *i*th participant
*x*_*pt*_: Assigned value for the RM used as PT item
*x*_*stb*_: Stability study mean
*z*_*i*_; * z′*_*i*_: *z*-Score and *z′*-score performance statistics
*σ*_*pt*_: Standard deviation for proficiency assessment

## References

[1] United Nations General Assembly. Resolution A/RES/64/292—The human right to water and sanitation. https://www.un.org/ga/search/view_doc.asp?symbol=A/RES/64/292 (2010).

[2] Nicaraguan Legislative Power. Ley general del medio ambiente y los recursos naturales de Nicaragua [Nicaraguan general law of environment and natural resources]. *La Gaceta.* **105**, Managua (1996).

[3] Nicaraguan Legislative Power. Política nacional de los recursos hídricos [National policy of water resources]. *La Gaceta.* **233**, Managua (2001).

[4] Nicaraguan Legislative Power. Ley general de aguas nacionales [General law of national water]. *La Gaceta.* **169**, Managua (2007).

[5] Centro de Investigación para los Recursos Acuáticos. https://www.ciraunan.edu.ni (2017).

[6] PTB. Improvement of the services that ensure the quality in the water sector of Nicaragua. https://www.ptb.de/cms/fileadmin/internet/fachabteilungen/abteilung_q/q.5_technische_zusammenarbeit/projektprofile/PTB_project_Nicaragua_95253_SP.pdf (2017).

[7] NTON 05 007–98 Norma para la clasificación de los recursos hídricos. *La Gaceta*. **30**. Nicaraguan Legislative Power, Managua (2000).

[8] International Organization for Standardization. General requirements for the competence of testing and calibration laboratories (ISO/IEC 17025) (2005).

[9] BIPM, IEC, IFCC, ILAC, ISO, IUPAC, IUPAP & OIML. Evaluation of measurement data – Guide to the expression of uncertainty in measurement GUM: 1995 with minor corrections. *Joint Committee for Guides in Metrology, JCGM*, 100. http://www.bipm.org/utils/common/documents/jcgm/JCGM_100_2008_E.pdf (2008).

[10] International Organization for Standardization. Water quality—Determination of pH (ISO 10523) (2008).

[11] Metrohm Competence Center Titration. Chloride titrations with potentiometric indication. Application Bulletin 130/3e. https://partners.metrohm.com/GetDocument?action=get_dms_document&docid=1346601 (n.d.).

[12] International Organization for Standardization. Water quality—Determination of dissolved anions by liquid chromatography of ions—Part 1: Determination of bromide, chloride, fluoride, nitrate, nitrite, phosphate and sulfate (ISO 10304-1) (2007).

[13] Khatri P, Gupta KK, Gupta RK, Panchariya PC (2021). Towards the green analytics: Design and development of sustainable drinking water quality monitoring system for Shekhawati Region in Rajasthan. J. Metrol. Soc. India MAPAN.

[14] International Organization for Standardization. Conformity assessment—General requirements for proficiency testing (ISO/IEC 17043) (2010).

[15] International Organization for Standardization. Statistical methods for use in proficiency testing by interlaboratory comparisons (ISO 13528) (2015).

[16] Thompson M, Ellison SL, Wood R (2006). The international harmonized protocol for the proficiency testing of analytical chemistry laboratories (IUPAC Technical Report). Pure Appl. Chem..

[17] International Organization for Standardization. General requirements for the competence of reference material producers (ISO Guide 34) (2009).

[18] International Organization for Standardization. Reference materials—General and statistical principles for certification (ISO Guide 35) (2006).

[19] Buck R (2002). Measurement of pH—Definition, standards, and procedures (IUPAC Recommendations 2002). Pure Appl. Chem..

[20] Kuselman I, Fajgelj A (2010). IUPAC/CITAC guide: Selection and use of proficiency testing schemes for a limited number of participants—Chemical analytical laboratories (IUPAC Technical Report). Pure Appl. Chem..

[21] Microsoft. Microsoft Excel [version 4.0.7177.5000]. Microsoft, Redmond, Washington (2010).

[22] R Core Team. R: A language and environment for statistical computing [version 3.1.1]. R Foundation for Statistical Computing, Vienna, Austria. http://www.R-project.org/ (2014).

[23] International Organization for Standardization. Reference materials—Guidance for characterization and assessment of homogeneity and stability (ISO Guide 35) (2017).

[24] Armishaw, P., King, B., & Millar, R. Achieving traceability in chemical measurement—A metrological approach to proficiency testing. In: De Bièvre P., Günzler H. (eds) *Traceability in Chemical Measurement*. 114–120. 10.1007/3-540-27093-0_19 (2003).

[25] Koepke A, Lafarge T, Possolo A, Toman B (2017). Consensus building for interlaboratory studies, key comparisons, and meta-analysis. Metrologia.

[26] EURACHEM & CITAC. Eurachem/CITAC Guide CG4: Quantifying Uncertainty in Analytical Measurement. 3rd edition, ISBN 978-0-948926-30-3 (2012). https://www.eurachem.org/images/stories/Guides/pdf/QUAM2012_P1.pdf

[27] Thompson, M. The amazing Horwitz function. *R. Soc. Chem. AMC Tech. Brief*. **17**. (2004). http://www.rsc.org/images/horwitz-function-technical-brief-17_tcm18-214859.pdf

[28] LACOMET. Informe final de resultados LACOMET 00013217—Ensayo de aptitud LACOMET-DMQ-001-2017 Conductividad y pH (en disolución acuosa a 25 °C). https://drive.google.com/file/d/1PupqdX21C9v9jnNbUzpemL0gQOmHwnWB/view (2017).

[29] LACOMET. Reporte para los participantes—Ensayo de aptitud LACOMET-DMQ-002-2013 Actividad del ion hidronio, pH, a 25 °C. https://drive.google.com/file/d/1PupqdX21C9v9jnNbUzpemL0gQOmHwnWB/view (2013).

[30] Snedecor G, Cochran W (1989). Statistical Methods.

[31] International Organization for Standardization. Accuracy (trueness and precision) of measurement methods and results—Part 2: Basic method for the determination of repeatability and reproducibility of a standard measurement method (ISO 5725-2) (1994).

[32] CIRA/UNAN-Managua. Reunión de cierre y presentación del Informe Final de la Prueba Interlaboratorio: Determinación de Conductividad eléctrica, Dureza total, Dureza cálcica, Calcio y Magnesio en disolución acuosa. Retrieved from http://www.cira.unan.edu.ni/index.php/reunion-cierre-presentacion-del-informe-final-la-prueba-interlaboratorio-determinacion-conductividad-electrica-dureza-total-dureza-calcica-calcio-magnesio-disolucion-acuosa/ (2018).

