# Supplementary material for: Improving the quality control of drinking water in Nicaragua through proficiency testing in a metrological multilateral cooperation project
Source: Sci Rep. 2021 Aug 19;11:16853. doi: 10.1038/s41598-021-96230-w (PMC8377044; doi:10.1038/s41598-021-96230-w)
Supplement: Supplementary file 1 — Supplementary Information. [file 41598_2021_96230_MOESM1_ESM.docx]

**SUPPLEMENTARY INFORMATION**

**Improving the quality control of drinking water in Nicaragua through proficiency testing in a metrological multilateral cooperation project**

Gabriel Molina-Castro^1*^, Jimmy Venegas-Padilla^1^, Junette Molina-Marcia^3^, Luciana Scarioni^2^, Bryan Calderón-Jiménez^1*^

^1^ Chemical Metrology Division, Costa Rican Metrology Laboratory, San Jose, Costa Rica,

^2^ By order of Physikalisch-Technische Bundesanstalt, Braunschweig, Germany,

^3^ Laboratory of Natural Water, Research Center for Aquatic Resources of Nicaragua, Managua, Nicaragua

**Table S1.** Participation of testing laboratories involved in the two stages of the cooperation project.

| **Laboratory Name** | **First stage** | | **Second stage** | |
| --- | --- | --- | --- | --- |
|  | **Workshops** | **PT-1** | **Workshops** | **PT-2** |
| CIRA/UNAN | 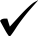 | 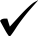 | 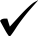 | 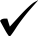 |
| PIENSA - UNI | 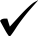 | 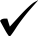 | 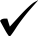 | 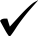 |
| CIDEA/UCA | 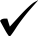 | 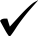 | 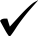 | 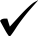 |
| CETEAL - UNI | 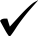 | 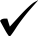 | 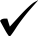 | 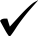 |
| ENEL | 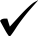 | 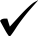 | 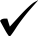 | 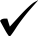 |
| MEM | 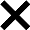 | 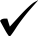 | 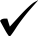 | 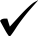 |
| UNAN/León | 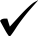 | 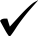 | 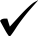 | 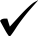 |
| Bengoechea | 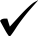 | 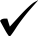 | 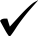 | 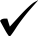 |
| LAQUISA | 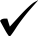 | 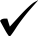 | 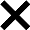 | 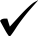 |
| ENACAL - León | 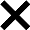 | 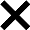 | 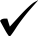 | 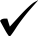 |
| 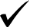: Participation 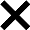: No participation | | | | |

**Table S2.** Summary of gravimetric preparation of the RM batches.

| **PT** | **Reference**  **Material ID** | **Chemical reagents** | **Starting material mass (g)** | **Final mass solution (g)** | **Final concentration**  **(mg kg^-1^)** | **Expanded uncertainty, *U* (*k* = 2)** |
| --- | --- | --- | --- | --- | --- | --- |
| PT-1 | An-02 (Cl^-^) | NaCl | 0.4912 | 3999.660 | 74.00 | 0.13 |
|  | An-06 (NO_3_^-^) | NaNO_3_ | 0.2604 | 3999.985 | 47.22 | 0.08 |
|  | pH-00 | Na_2_B_4_O_7_·10H_2_O | 15.0916 | 3955.180 | - | - |
| PT-2 | An-02 (Cl^-^) | NaCl | 0.859 65 | 6011.112 | 86.75 | 0.10 |
|  | An-06 (NO_3_^-^) | KNO_3_ | 0.149 55 | 3009.700 | 30.473 | 0.096 |
|  | pH-00 | C_8_H_5_KO_4_ | 30.6476 | 3000.011 | - | - |

**Table S3.** IC system operating conditions used for Cl^-^ and NO_3_^-^ measurements.

| **Operating conditions** | |
| --- | --- |
| Column: | Metrosep A Supp 5 150/4.0 |
| Eluent: | 3.2 mM Na_2_CO_3_ / 1.0 mM NaHCO_3_ |
| Flow eluent: | 0.7 mL min^-1^ |
| Chemical suppressor: | 0.1 mol L^-1^ H_2_SO_4_ |
| Oven temperature: | 30 °C |
| Injection volume: | 20 µL |
| Detector: | Conductivity |
| Mobile phase: | 3.2 mmol L^-1^ Na_2_CO_3_ / 1.0 mmol L^-1^ NaHCO_3_ |

**Table S4.** Assigned values, metrological traceability sources and legislation requirements for the PT items.

| **Parameter** | **Assigned values** | | **Nicaraguan legislation** | |
| --- | --- | --- | --- | --- |
|  | **PT-1** | **PT-2** | **Recommended**  **value** | **Maximum**  **permissible** |
| Cl^-^ (mg kg^-1^) | 74.0 | 86.0 | 25 | 250 |
| NO_3_^-^ (mg kg^-1^) | 46.8 | 30.4 | 25 | 50 |
| pH (1) | 9.17 | 4.03 | 6.5 to 8.5 | ND |

ND: Not defined.

|  | **(a) Uncertainty Intervals** | **(b) Unilateral Degrees of Equivalence** |
| --- | --- | --- |
| **NO_3_^-^ (PT-1)** | 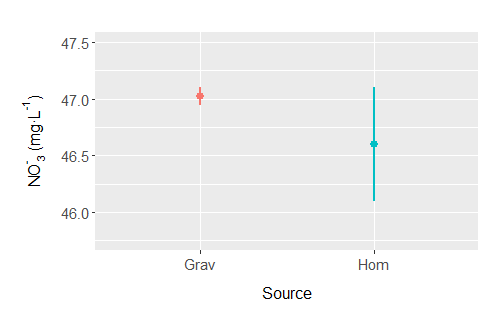 | 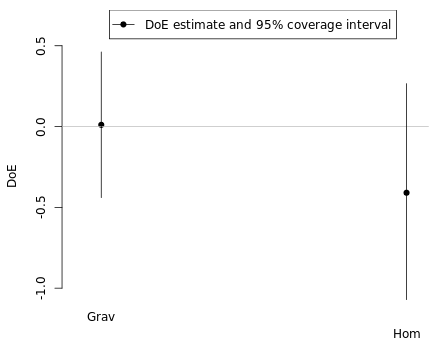 |
| **Cl^-^ (PT-1)** | 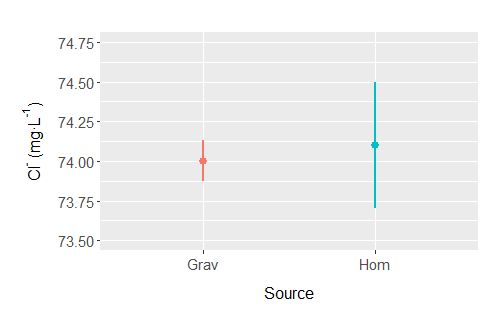 | 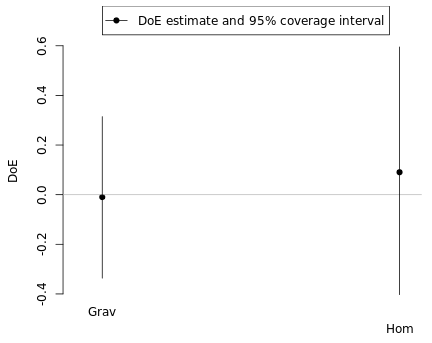 |
| **NO_3_^-^ (PT-2)** | 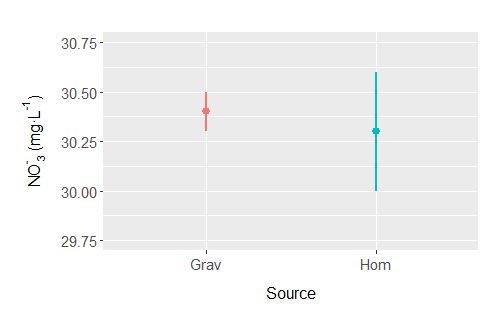 | 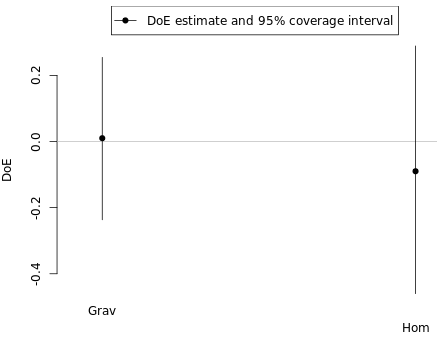 |
| **Cl^-^ (PT-2)** | 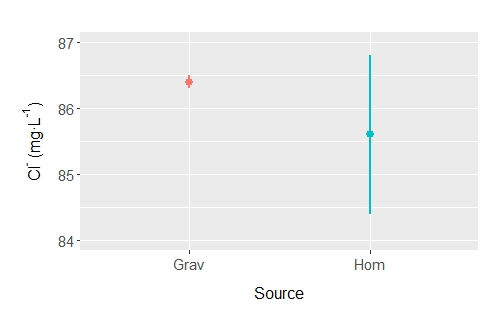 | 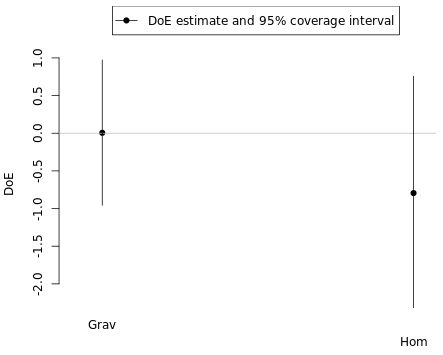 |

**Figure S1.** Agreement analysis between *x_grav_* and *x_hom_* values for NO_3_^-^ and Cl^-^ using **(a)** uncertainty intervals comparison and **(b)** unilateral degrees of equivalence estimation.


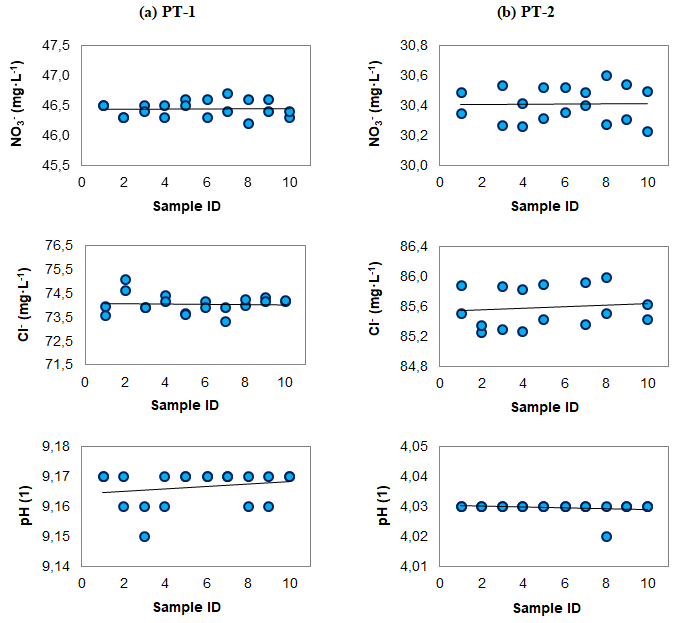


**Figure S2.** Homogeneity study results and absence of trends by preparation (solid black line) for columns **(a)** PT-1 RMs and **(b)** PT-2 RMs.

**
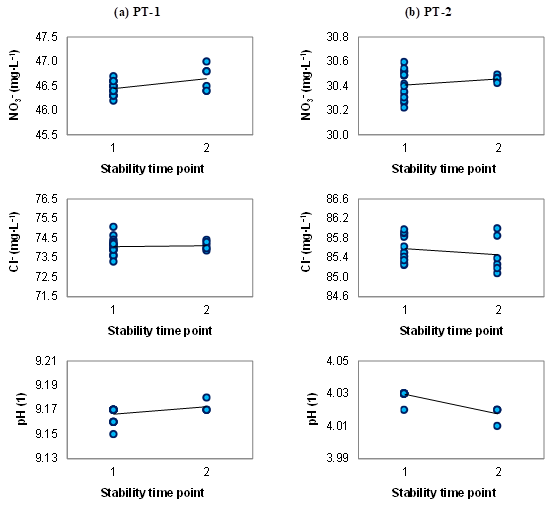
**

**Figure S3.** Stability study results for columns **(a)** PT-1 RMs and **(b)** PT-2 RMs.

**Figure S4.** Kernel density plots of the observed distributions of the participants’ results and the assigned values and their estimated expanded uncertainties for columns **(a)** PT-1 RMs and **(b)** PT-2 RMs.
